# Effects of acute mental stress on conditioned pain modulation in temporomandibular disorders patients and healthy individuals

**DOI:** 10.1590/1678-7757-2020-0952

**Published:** 2021-06-04

**Authors:** Dyna Mara Araújo Oliveira FERREIRA, Yuri Martins COSTA, Leonardo Rigoldi BONJARDIM, Paulo César Rodrigues CONTI

**Affiliations:** 1 Universidade de São Paulo Faculdade de Odontologia de Bauru Departamento de Prótese e Periodontia Bauru Brasil Universidade de São Paulo, Faculdade de Odontologia de Bauru, Departamento de Prótese e Periodontia, Bauru, Brasil.; 2 Universidade de Campinas Faculdade de Odontologia de Piracicaba Departamento de Biociências Piracicaba Brasil Universidade de Campinas, Faculdade de Odontologia de Piracicaba, Departamento de Biociências, Piracicaba, Brasil.; 3 Universidade de São Paulo Faculdade de Odontologia de Bauru Departamento de Ciências Biológicas Bauru Brasil Universidade de São Paulo, Faculdade de Odontologia de Bauru, Departamento de Ciências Biológicas, Bauru, Brasil.

**Keywords:** Psychological stress, Neural inhibition, Pain threshold, Temporomandibular joint dysfunction syndrome

## Abstract

Stress is a contributing factor to painful temporomandibular disorders (TMD). Nevertheless, the underpinnings of this relationship are not fully understood.

**Objective:**

To investigate the effects of acute mental stress on conditioned pain modulation (CPM) in TMD patients compared with healthy individuals.

**Methodology:**

Twenty women with chronic myofascial TMD diagnosed according to the RDC/TMD and 20 age-matched healthy women had the CPM assessed before and after a stressful task using the Paced Auditory Serial Addition Task (PASAT) in a single session. Subjective stress response was assessed with the aid of visual analog scale (VAS). Pressure pain threshold (PPT) on masseter muscle was the test stimulus (TS) and immersion of the participant’s hand on hot water was the conditioning stimulus (CS) - CPM-sequential paradigm.

**Results:**

Healthy individuals reported PASAT are more stressful when compared with TMD patients and the stress task did not affect the CPM in neither group. Nonetheless, a negative correlation was observed between change in CPM and change in TS from baseline to post-stress session, which indicates that the greater the increase in PPT after the stress task, the greater was the decrease in CPM magnitude. The correlation was strong for healthy controls (r=- 0.72, p<0.001) and moderate for TMD patients (r=- 0.44, p=0.047).

**Conclusions:**

The correlation between the change in CPM and the TS change following the stress task may possibly indicate an overlapping pathway between stress-induced analgesia/hyperalgesia and descending pain inhibition.

## Introduction

Pain-related temporomandibular disorders (TMD) are the most common chronic orofacial pain disorders.^1,2^ Pain-related TMD negatively affect the quality of life, since the pain can interfere with daily activities, such as talking, eating, laughing and working.^3^ Several studies have investigated the relationship between TMDs and stress, i.e., a perception of uncontrollably and unpredictability of stimuli.^4^ TMD patients present greater psychosocial stress levels and report higher number of traumatic stressors when compared with pain-free subjects.^5,6^ Moreover, traumatic stressors are related to increased pain severity, distress, and disability among patients with TMD,^5^ and the Orofacial Pain Prospective Evaluation and Risk Assessment (OPPERA) cohort study observed that psychosocial stress is a risk factor for the onset of pain-related TMD.^7^ Finally, dysfunction of sympathetic nervous system and hypothalamic-pituitary-adrenal axis in some patients with TMD have also been demonstrated.^8,9^ Therefore, there is evidence in favor of a relevant relationship between stress and TMD. Nevertheless, the underpinnings of this relationship are not fully understood.

Few studies have investigated the effects of experimental stress on pain sensitivity in TMD patients.^8,10,11^ Pressure and thermal hyperalgesia of the masseter region following experimental stress have been reported in case-control investigations.^10,11^ On the other hand, there is no effect of stress on ischemic pain sensitivity of the arm.^8^ More recently, impairment of endogenous pain inhibitory system has been suggested as contributing factor for increased pain sensitivity in TMD patients.^12-14^ Thus, it might be expected that stress may interfere with the endogenous pain inhibitory capacity. The conditioned pain modulation (CPM) paradigm can assess the endogenous pain inhibition in humans and corresponds to the diffuse noxious inhibitory control (DNIC) that was first described in rats.^15,16^ The CPM encompasses the evaluation of a painful test stimulus (TS) in the absence and in the presence (or subsequently) of a second painful stimulus applied to a remote region of the body, i.e., the conditioning stimulus (CS).^17^ Nonetheless, the relationship between stress and CPM in TMD patients has not been properly addressed in the literature. The possible interaction between stress and CPM in pain-related TMD patients is of clinical importance considering the diagnostic and treatment implications of pain modulation impairment in chronic orofacial pain.^15^

Thus, the primary objective of our study was to assess the effect of acute mental stress in the CPM magnitude of TMD patients and healthy individuals. We hypothesized that there would be a significant effect of the experimental stress in the CPM magnitude of TMD patients and healthy individuals.

## Methodology

### Participants

A total of 20 healthy women and 20 women with myofascial TMD were selected from the local community through advertisements at Bauru School of Dentistry. Only female participants were included to control the influence of gender on pain perception.^18^The examination followed the Research Diagnostic Criteria for Temporomandibular Disorders (RDC/TMD)^19^ and was performed by an orofacial pain specialist (DMAOF). The inclusion criteria for the TMD group were as follows: (a) adults between 18 and 50 years of age; (b) myofascial pain with or without jaw opening limitation;^19^ (c) pain present for at least 3 months; (d) no TMD treatment in the previous 3 months; (e) no concurrent pain or diagnosis of other painful conditions (e.g., pulpal and periodontal pain, fibromyalgia, chronic widespread pain or irritable bowel syndrome). Inclusion for the control group was absence of any complaint or diagnosis of pain syndrome at the time of study enrollment. Exclusion criteria were: (a) the presence or history of injury in the testing site (face); (b) systemic conditions (e.g., diabetes, cardiovascular or inflammatory disorders); (c) current use of drug targeting the central nervous system (e.g., muscle relaxants, anticonvulsants, antidepressants and anxiolytics); (d) psychiatric disorders (e.g., anxiety, depression, bipolar disorder); (e) pregnancy. The exclusion criteria were assessed based on a detailed medical history. Moreover, all subjects were required to refrain from analgesic intake 48 hours before the experimental session. Written consent from each subject for study participation was obtained after a full explanation of the procedures. The study was conducted in accordance with the Declaration of Helsinki and approved by the Human Research Ethics Committee of the Bauru School of Dentistry, University of São Paulo.

### Design

**Figure 1:**
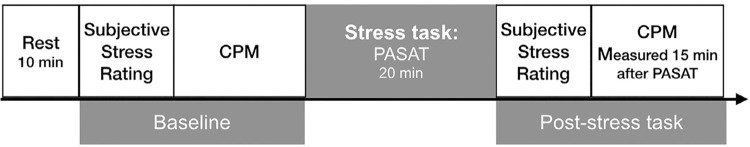
Experimental procedure: after resting for 10 min, subjective stress and CPM (conditioned pain modulation test) were measured. The subjective stress was rated on an analogic visual analog scale (0 = no stress and 10 = most intense stress imaginable). Sequential CPM protocol was performed by applying pressure pain threshold on masseter muscle as test stimulus and immersion of hand in hot water bath as conditioning stimulus. Two trial of PASAT was followed by post-stress task measures

This case-control study was performed in one session and CPM was assessed before and after an acute mental stress task. All sessions were conducted in a quiet room with a constant temperature of 25°C±1°C between 1:00 pm and 4:00 pm. The participants were asked to avoid the use of tobacco and ingestion of beverages or food 30 minutes before the procedures. Following 10 minutes of resting in the room, participants had the subjective stress and CPM assessed. Subsequently, the stress task was performed with two trials of Paced Auditory Serial Addition Task (PASAT) to maintain a high level of stress (20 minutes). Immediately after, subjective stress level was reassessed. It has been demonstrated that HPA axis stress response influences pain sensitivity ^20^ and that high cortisol levels have been found from 10 to 20 min after PASAT.^21^ To avoid possibly missing a cortisol effect on pain sensitivity, CPM was measured around 15 minutes after the end of the stress task. Blinding assessments were not possible, considering that the first author applied the stress task and collected the data. Prior the procedures, demographic and psychological characteristics of our sample were collected and has been reported elsewhere.^11^ The study design is illustrated in Figure 1.

### Stress task

To induce acute mental stress, PASAT was applied. The PASAT encompasses mental arithmetic tasks, in which numbers are auditorily presented and the participant must sum consecutive numbers in sets of two, adding only two numbers at a time.^22^ The participant was told to give her answer verbally before the presentation of the next number for a response to be scored as correct. The single numbers were presented at intervals of 1.2, 1.6, 2.0 and 2.4 seconds and one trial lasted approximately 600 seconds.^11^ The PASAT has been shown to be an effective acute psychological stressor.^23,24^

Before the stress task, the participant was told that the average performance is 70-80% of right answers and that her individual performance would be compared to other participants’ performance. During the trial, the investigator remained in the room with the participant and pretended to take notes on the subject’s answers. Once the trial was over, the investigator informed the participant about her negative performance (below the average) and, asked her to try a second trial to improve her performance. All participants received negative feedback. Following a similar second trial, the participant was again told that she scored below average and the stress task was terminated. Two trials of PASAT were applied to increase the stress response and, thus, the full task took approximately 20 minutes to be completed.^11^

### Subjective stress response

The subjective stress response was assessed by a visual analog scale (VAS). The VAS consisted of a 10-cm line with 2 anchor points at its extremes, set as 0 = no stress and 10 = most intense stress imaginable. The participants were asked to rate their subjective stress level at baseline and immediately after stress task.

### Conditioned pain modulation

A CPM-sequential paradigm was performed by using the pressurize pain threshold (PPT) on masseter muscle as test stimulus (TS) and immersion of the participant’s hand on hot water as conditioning stimulus (CS). This protocol has been used in our previous studies.^12,25^ The PPT was assessed in triplicates using a digital pressure algometer (Kratos) fitted with a probe (1 cm^2^surface area and flat circular-shaped tip) placed over the test site. The test site was defined as the most painful masseter spot according to self-report for TMD patients and the masseter body ipsilateral to the dominant hand for healthy individuals. Following this, the CS was applied at the contralateral body side and consisted of immersion of the participant’s hand up to the wrist for 1 minute on hot water bath at 46 °C±1°C, controlled by a thermostat. During the hand immersion the subjects were asked to rate the pain intensity on a numerical rate scale (NRS) ranging from 0=no pain to 100=the most intense pain imaginable. The CS pain intensity was maintained>30 NRS for all participants^17^. Immediately after the participants removed their hand from the water, then PPT was again assessed at the masseter muscle. CPM was estimated as percent change in PPT after the CS relative to PPT before the CS. Pain inhibition along the protocol was represented by a negative value, whereas pain facilitation was denoted by a positive value.^17^

### Statistics

It was expected that a medium effect size f of 0.45 for CPM differences would be worth detecting considering the interactions from ANCOVA with one within-subject factor, one between-subject factor, one continuous covariate, 80% power and a a 5% significance level. Therefore, the sample size estimation was at least 20 subjects per group.

The outcomes were presented as mean ± standard deviation (SD) and 95% confidence interval of the mean (95% CI) unless otherwise noticed. Normal distribution was assessed with the aid of Q-Q plots and log_10_ transformations were applied for the continuous variables when relevant departures from normality were observed. Thus, the subjective stress rating was log_10_ transformed. Normality was then re-assessed, and the transformed variable was considered normally distributed.

Mixed design ANCOVA was computed to assess CPM differences considering one between-subject factor, group–2 levels (TMD patients and healthy individuals), one within-subject factor, session–2 levels (baseline and post-stress), and one continuous covariate, i.e., D-PPT (percent change in the PPT from baseline to post-stress task). Pairwise *post-hoc* comparison analyses were performed using Tukey’s Honestly Statistical Difference (HSD). The significance level was set at 5% (p=0.050). Moreover, Pearson product-moment correlation was computed to evaluate the association between the D-CPM (percent change in the CPM from baseline to post-stress task) and D-PPT. The strength of correlation was evaluated based on the r coefficient, and the following cut-offs were applied: small (r<0.39), moderate (r>0.4 and <0.69) or strong (r>0.69) correlation.^26^

Likewise, mixed design ANOVA was computed to assess differences in the perceived stress considering one between-subject factor, group–2 levels (TMD patients and healthy participants), one within-subject factor, session–2 levels (baseline and post-stress task). Pairwise *post-hoc* comparison analyses were performed using Tukey’s Honestly Statistical Difference (HSD).

## Results

Baseline demographics and psychological characteristics of TMD and healthy individuals are reported elsewhere.^11^ In short, there were no significant differences in age, anxiety and perceived stress at baseline between groups (p>0.050). Mean age was 30.1 years (SD 9.1) for TMD patients and 29.5 (6.7) for healthy individuals. Likewise, mean trait-anxiety was 39 (SD 9.7) and 38.6 (SD 9.6), mean state-anxiety was 35.6 (SD 7.5) and 32 (SD 7.3) and perceived stress over past month was 21.6 (SD 7.2) and 23.5 (SD 8.2), respectively for TMD patients and healthy individuals. However, pain catastrophizing was higher in TMD patients (22.9; SD 14.2) when compared with healthy individuals (12.3; SD 9.9).^11^

The stress-task significantly increased the subjective stress values throughout the sessions in both groups (F_1,37_=59.1, p<0.001 and partial η^2^=0.61). Moreover, post-stress task values were higher for healthy individuals when compared with TMD patients (Tukey’s: p=0.0013). The mean (95% CI) of the subjective stress differences from baseline to post-stress task for the TMD patients and healthy individuals were, respectively, 1.5 (95% CI = 0.48 to 2.56) and 3.5 (95% CI=2.59 to 4.51).

**Figure 2:**
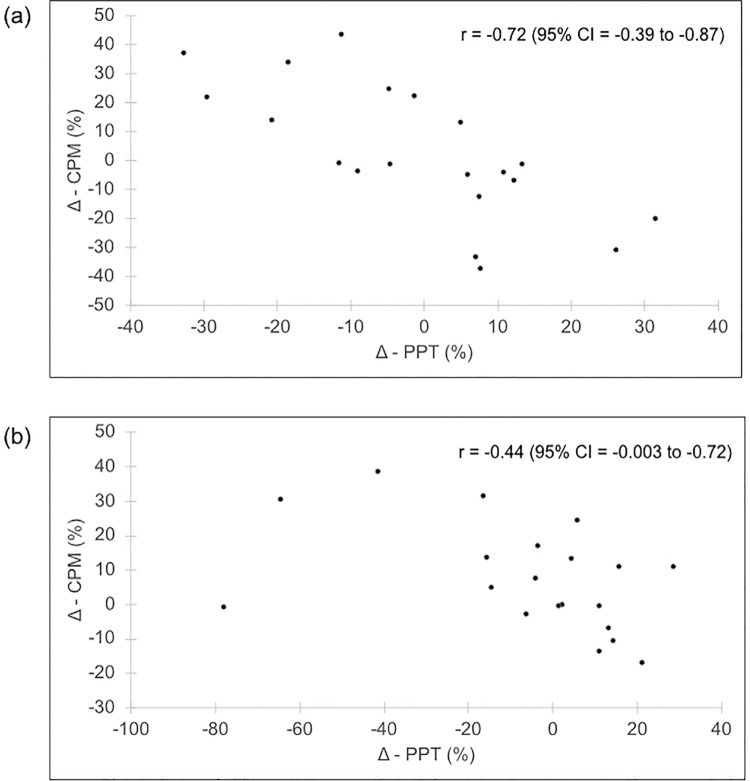
A scatter plot showing the negative correlation between the relative change in conditioned pain modulation (Δ-CPM) and relative change in pressure pain threshold (Δ-PPT) from baseline to post-stress session in (a) healthy individuals and (b) temporomandibular disorders (TMD) patients

Table 1 shows the CPM values at baseline and post-stress task sessions for TMD patients and healthy individuals. There were no significant main effects of neither group (F_1, 37_=0.02, p=0.882 and partial l^2^= 0.00) nor session (F_1, 37_ =1.61, p=0.211 and partial l^2^= 0.04) in the CPM magnitude. Likewise, there was no significant interaction between group and session (F_1, 37_=0.22, p=0.634 and partial l^2^=0.00). Nonetheless, there was a significant interaction between session and the continuous covariate D-PPT (F_1, 37_=14.4, p<0.001 and partial l^2^=0.28). Thus, the D-CPM was significantly associated with D-PPT (Figure 2). There was a strong negative correlation between the D-CPM and D-PPT for healthy individuals (r=-0.72, p<0.001) and a moderate negative correlation between the D-CPM and D-PPT for TMD patients (r=-0.44, p=0.047) (Figure 2).

**Table 1:** Subjective stress rating, pressure pain threshold (PPT) as test stimulus and conditioned pain modulation (CPM) at baseline and post-stress task session in healthy subjects and patients with temporomandibular disorders (TMD)

	Baseline	Post-stress task
	**Mean (SD)**	**Mean (SD)**
**Healthy (n=20)**		
Subjective stress rating (0-10 cm)	1.61 (1.97)	5.16 (1.89)
PPT (kgf/cm^2^)	1.13 (0.35)	1.10 (0.27)
CPM (%)^a^	- 8.8 (21.31)	- 6.4 (22.11)
**TMD (n=20)**		
Subjective stress rating (0-10 cm)	1.24 (1.60)	2.77 (1.83)
PPT (kgf/cm^2^)	0.90 (0.29)	0.91 (0.26)
CPM (%)^a^	-10.2 (17.92)	- 2.9 (16.69)

^a^CPM negative values mean pain inhibition along the protocol.

## Discussion

Our study investigated the effects of acute mental stress in the CPM magnitude of TMD patients and healthy controls. In general, the experimental stress had no effect on the CPM magnitude. However, CPM changes (D-CPM) were associated with changes on the TS (D-PPT), in which the greater the increase in TS after stress task the greater the decrease in CPM magnitude.

The effects of acute mental stress on CPM have been investigated in healthy individuals and chronic pain patients, such as headache, fibromyalgia and whiplash associated disorders.^27-32^ To the best our knowledge, this is the first study that evaluated the effects of stress on CPM in TMD patients. Acute mental stress had no direct effect on CPM neither in healthy individuals nor in TMD patients. Our results are in agreement with both Hoegh, et al.^31^ (2020) and Cathcart, et al.^27^ (2010), who also reported that stress manipulation did not significantly change the CPM in neither healthy individuals nor tension-type headache patients. On the other hand, reduced CPM after and during stress has been demonstrated in healthy individuals, with heat pain as the TS.^29,30,32^ These findings support the somatosensory modality influences on CPM.^31,32^ Thus, it could be argued that the stress effect on CPM can be more easily observed when a superficial nociceptive input, e.g., heat, is applied instead of a deep nociceptive input, e.g., pressure. Nonetheless, PPT is reported to be the most reliable TS^33^ and it has been frequently applied to assess endogenous pain inhibitory mechanisms in the craniofacial region.^13,34^

Methodological issues may also explain the absence of a significant effect of stress on CPM. For instance, a single CPM trial could not be sufficient to detect mild effects on pain inhibitory system induced by stress in the trigeminal region. Nahman-Averbuch, et al.^35^ (2013) showed that migraine patients had a CPM response similar to controls during the first CPM trial. However, migraine patients failed to sustain their CPM response along repetitions of the CPM-parallel paradigm. Moreover, there is also evidence that suggests a weaker CPM effect when the TS is applied in trigeminally innervated areas.^36^ Therefore, repeated CPM trials and comparison between spinal and trigeminal innervation are recommended in future investigations of the stress effects on the endogenous pain modulation.

Although there were no significant stress influences on CPM, a decrease in CPM magnitude after stress task was correlated with the PPT change from baseline to post-stress task session. Specifically, the greater the increase in PPT after stress, the greater was the decrease in CPM magnitude, which indicates a waning CPM effect. Stress can exert bidirectional modulatory effects on pain sensitivity, either reducing (stress-induced analgesia) or exacerbating the pain (stress-induced hyperalgesia).^37,38^ Our findings suggest that when acute mental stress reduces the TS painfulness, i.e., stress-induced analgesia, the CS seems to less vigorously engage the descending pain inhibitory system. Indeed, a functional magnetic resonance imaging study showed that stress-induced analgesia and CPM paradigm activate similar brain regions such as insula, dorsal and rostral anterior cingulate cortex.^39^ Moreover, there is evidence for a role of the opioid and serotonergic system in stress-induced analgesia and descending pain inhibition.^37,40^ Therefore, considering the overlap between these two types of endogenous analgesia, a reduced CPM magnitude following the stress task could be partially attributed to a previous activation of inhibitory system. Nonetheless, given the limited sample size, the negative relation between change in CPM and change in PPT from baseline to post-stress task session should be interpreted with caution.

This case-control study has some limitations that need to be addressed. First, we only used a subjective measurement as the stress response. Second, we only investigated female participants, so generalizability is reduced. Finally, the sample size (n = 40) was small to build robust regression models in which the effect of additional covariates and factors could be analyzed. Therefore, further investigations are necessary to confirm our findings about the relationship between stress and CPM in TMD patients.

Thus, considering the limitations of our study, our findings indicate a moderate to strong correlation between the CPM and acute mental stress possibly due to an effect modifier of the stress task on the TS. Accordingly, stress-induced analgesia/hyperalgesia and descending pain inhibition may possibly present overlapping pathways.
